# Artificial Neural Networks for Forecasting Passenger Flows on Metro Lines

**DOI:** 10.3390/s19153424

**Published:** 2019-08-05

**Authors:** Mariano Gallo, Giuseppina De Luca, Luca D’Acierno, Marilisa Botte

**Affiliations:** 1 Department of Engineering, University of Sannio, piazza Roma 21, 82100 Benevento, Italy; 2 Department of Civil, Architectural and Environmental Engineering, Federico II University of Naples, via Claudio 21, 80125 Naples, Italy

**Keywords:** artificial neural networks, metro, transportation, user flow forecast

## Abstract

Forecasting user flows on transportation networks is a fundamental task for Intelligent Transport Systems (ITSs). Indeed, most control and management strategies on transportation systems are based on the knowledge of user flows. For implementing ITS strategies, the forecast of user flows on some network links obtained as a function of user flows on other links (for instance, where data are available in real time with sensors) may provide a significant contribution. In this paper, we propose the use of Artificial Neural Networks (ANNs) for forecasting metro onboard passenger flows as a function of passenger counts at station turnstiles. We assume that metro station turnstiles record the number of passengers entering by means of an automatic counting system and that these data are available every few minutes (temporal aggregation); the objective is to estimate onboard passengers on each track section of the line (i.e., between two successive stations) as a function of turnstile data collected in the previous periods. The choice of the period length may depend on service schedules. Artificial Neural Networks are trained by using simulation data obtained with a dynamic loading procedure of the rail line. The proposed approach is tested on a real-scale case: Line 1 of the Naples metro system (Italy). Numerical results show that the proposed approach is able to forecast the flows on metro sections with satisfactory precision.

## 1. Introduction

Knowledge of user flows on transportation systems is crucial for implementing control and management policies. In this context, monitoring systems assume a central role and are widely used in many road and rail networks: they are one of the most important (and necessary) components of Intelligent Transport Systems (ITSs).

Monitoring systems are based on sensors (or detectors) that measure some characteristics of flows and transmit them to a control room, where the relevant data are used for implementing control and management strategies. For instance, knowing traffic flows on a road network is useful for implementing flow-responsive traffic-signal systems, while the user loads on public transport vehicles can be used for real-time scheduling/rescheduling tasks.

Despite their usefulness, monitoring systems are not always provided on road and rail networks or, sometimes, implemented systems do not have enough sensors to collect the data required for control and management strategies. Indeed, the costs of these systems require significant investments from public administration or public transport firms which are often inadmissible.

In this paper, we focus on metro lines, where access is controlled by turnstiles which can count the passengers entering each station with or without identifying their direction (in most of the existing stations, except terminals, passengers can board trains in different directions). These data can be easily collected every few minutes (e.g., fifteen-minute intervals) without installing new sensors. The objective of this paper is to propose a method based on Artificial Neural Networks (ANNs) for estimating the number of passengers on each segment of a metro line using data obtained from turnstiles. Indeed, on-board data are rarely available in real time because they require infrared scanners, or alternatively weight-based, image-based or photocell-based sensors that are seldom installed on coaches.

The paper is organised as follows: Section 2 provides the background; Section 3 describes the problem to solve and the ANN approach; the method adopted for generating the data used in numerical tests is reported in Section 4; Section 5 describes the case study and the results; Section 6 concludes and identifies some research prospects.

## 2. Background

### 2.1. Artificial Neural Networks

The Artificial Neural Network (ANN) is a mathematical method that is widely used for reproducing several physical phenomena and forecasting the results of some actions on (or variations of) the parameters/variables of the system. ANNs are considered to be black-boxes since the functions and the relationships between inputs and outputs are hidden, not known and, generally, not interpretable.

Both the strengths and weaknesses of ANNs are related to their black-box approach. ANNs can reproduce a phenomenon or approximate a function without making the parameters explicit; moreover, once trained, they are able to give the results rapidly. On the other hand, trained ANNs are not extendible even to similar cases and work only if the boundary conditions do not change significantly.

ANNs have been widely studied elsewhere; they were initially introduced in [1,2,3] and then developed in other pioneering contributions [4,5,6,7,8]. Many general books focus on ANNs; here we refer to [9,10,11,12,13,14].

Literature reviews have been proposed in several papers. Scarselli and Tsoi [15] presented a review of studies that used Feedforward Neural Networks to approximate some functions, examining computational aspects, structures of the network (hidden layers and neurons), and training algorithms. They also proposed two training algorithms. Baptista and Morgado-Dias [16] examined the numerous software tools available, with the intention to help choose the most appropriate tool while considering its features (operating system, minimum hardware, kind of licence, algorithms implemented, and so on). Timotheou [17] reviewed random neural networks and their application to several problems. Extreme learning machines were reviewed in [18], while reviews on deep learning in neural networks can be found in [19,20]. Finally, here we refer to Yao [21] for an appraisal of evolutionary artificial neural networks.

### 2.2. Road Traffic Flow Forecasting

Two main types of transportation flow forecasting problems can be identified: (i) short-term forecasting and (ii) traffic data spatial extension. Some papers that applied ANNs to these problems were reviewed in [22].

The first problem aims to forecast the traffic flows (or user flows) that use a road section (or a transit line) in a future time interval, using the data measured in the previous time intervals in the same road section. This problem has been widely studied elsewhere, and a complete review would deserve a specific paper; here we refer to [23,24]. For solving this problem, several methods were proposed; in this paper, we focus on most of the literature that has used ANNs.

Kirby et al. [25] discussed the use of ANNs for forecasting traffic flows on motorways up to an hour ahead and compared this approach with other statistical models. Smith and Demetsky [26] compared the performances of ANNs with traditional methods for solving the short-term traffic flow prediction problem, such as data-based algorithms and time-series models; they found that the back-propagation neural network model was able to predict future traffic flows on highways better than the other models. In the same research field, ANNs were used for modelling freeway traffic in a macroscopic environment [27]; the authors found that the neural network model was able to capture the traffic dynamics quite closely and was “*computationally efficient for real-time implementation*”. ANNs were proposed as tools for predicting congestion and forecasting flows in [28]; the authors also discussed whether ANNs were able to estimate parameters that cannot be directly measured with road sensors. Park et al. [29] proposed a radial basis function neural network for short-term forecasting on freeways; they tested the method with real observations and compared it with other approaches such as Taylor series, single and double exponential smoothing methods, and back-propagation neural networks. Zheng et al. [30] proposed a Bayesian combined neural network approach for short-term forecasting on freeways, while a binary neural network was presented in [31]. Another application on a highway can be found in [32], while applications in urban environments can be found in [33,34]. Park et al. [35] used feedforward multilayer neural networks for estimating link travel times on freeways. Other applications of ANNs to short-term forecasting can be found in [36,37,38,39,40]. Ledoux [41] proposed the use of ANNs within an urban traffic flow model, while Florio and Mussone [42] studied traffic flow stability on freeways with neural network models.

Traffic data spatial extension problems have received less attention in the literature. Lin et al. [43] used a macroscopic model for short-term forecasting, which is also able to predict flows on other links. Zheng Zhu et al. [44] used ANNs for spatial extension of traffic flows at road intersections. Gallo and De Luca [45] proposed the use of ANNs for estimating traffic flows on some links of an urban road network according to the flows measured on other links.

Recently, deep learning methods have been proposed in the field of traffic prediction; a survey can be found in [46]. The authors identified four main deep learning models: deep neural networks (DNN), convolutional neural networks (CNN), recurrent neural networks (RNN), and deep reinforcement learning (referring, in particular, to the Deep Q-Network [47]). In the ITS field, among others, DNN, CNN and RNN are useful for time series prediction. However, the above methods have not yet been used for the spatial extension of traffic or passenger data.

Short-term traffic forecasting with deep learning was studied in [48], where a long short-term memory (LSTM) network was proposed; the method was tested on a case study in Beijing, showing promising forecast accuracy compared with other approaches. The same approach has also been used for traffic flow prediction with missing data in [49].

Temporal CNN was proposed in [50] for short-term forecasting of passenger demand, outperforming other models in test cases. Support vector machines and data denoising schemes were combined in [51] for traffic flow prediction; the proposed denoising algorithms improved the results in this hybrid model, compared to other approaches without a denoising strategy. Short-term travel speed prediction was studied in [52,53,54,55].

### 2.3. Metro Passenger Flow Forecasting

The specific problem tackled in this paper entails metro passenger flow prediction. The literature review in this field presents some interesting contributions.

Deep learning methods were proposed in [56] and tested on a Bus Rapid Transit (BRT) system. The proposed model forecasts the hourly flow, adopting a three-stage deep learning architecture. This paper also analyses the literature, identifying four different approaches: (1) traditional classical algorithms; (2) regressive models; (3) machine learning-based models, including ANNs; (4) hybrid models. All studied cases, however, refer to short-term or long-term time periods, without considering the spatial extension. Among them, cases reported in [57,58] are applied on railways and based on ANNs, focusing on short-term and long-term forecasting respectively. Short-term forecasting on urban metros was also studied along with other methods, such as Kalman filter [59] and ARIMA (autoregressive integrated moving average) models [60].

Li et al. [61] proposed a multiscale radial basis function (MSRBF) for forecasting short-term metro passenger flows on special occasions, such as sporting events, concerts, and so on. In this case, passenger flow is very irregular and predictions are more difficult to obtain. Ling et al. [62] used smart-card data for predicting passenger flows in the subway of Shenzhen (China); they analysed four predictive models: a historical average model, ANN, regression model and a gradient-boosted regression tree model. Liu et al. [63] proposed a deep learning method for short-term forecasting of metro inbound/outbound passenger flows, while Wang et al. [64] proposed a Novel Markov-Grey model for solving the same problem.

### 2.4. Contribution of the Paper

ANNs have been widely used in numerous scientific fields since the 1950s/1960s and in traffic engineering since the 1990s. Most applications in traffic engineering have focused on the temporal extent of data (more frequently short-term or, sometimes, long-term predictions) and road environments; fewer cases refer to transit systems. The spatial extent of data has been less widely studied and, to our best knowledge, the use of ANNs for the specific problem tackled in this paper has not been proposed elsewhere. Therefore, the originality of our contribution does not so much concern the method used, which is indeed consolidated, as the problem dealt with and the procedure used to construct the training datasets. Other more advanced methods, such as deep learning, will be the subject of further research, as will be discussed in the conclusions. In this paper, the performance of ANNs was not compared with other methods because there are no benchmarks. Indeed, almost all methods usually used as benchmarks in short-term forecasting problems are not applicable in our case, since they are time-series specific.

It is important to underline that the problem studied is relevant to the real-time management of metro lines. Data at turnstiles can be easily collected with methods that do not require significant additional investment, while data obtained through the above procedure (loads on line sections) are essential for service operators and, unlike the former, are not easily detectable in real time and continuously.

## 3. Problem Description and ANN Approach

**Figure 1:**
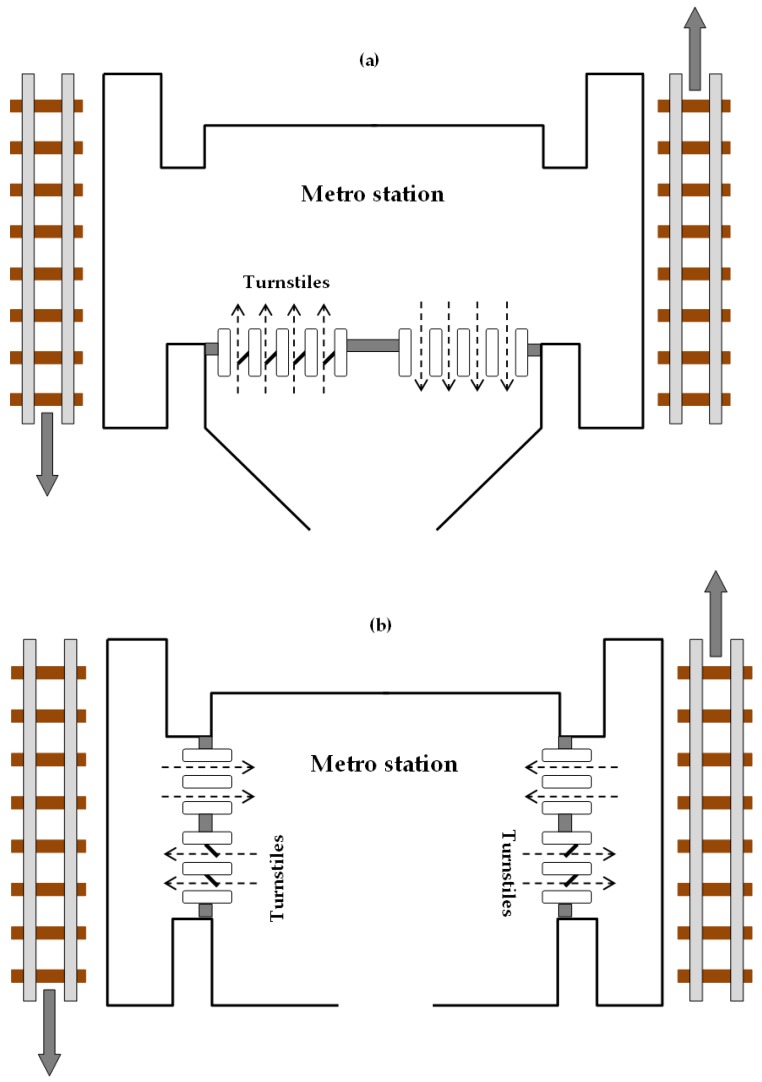
Types of metro stations: (**a**) turnstiles at the entrance; (**b**) turnstiles at platform accesses.

We assume that turnstiles control all accesses to a metro line: each user, entering a station, uses a ticket (or a pass) for crossing the turnstile. Moreover, the turnstiles are only able to count users entering the station without linking the origin of each trip with the corresponding destination. This situation is common to many metro lines, such as Line 1 of the Naples metro system (Italy) which will be the subject of the real-scale test. Indeed, turnstiles are often installed only for facilitating ticket control/validation and avoiding no-ticket trips, and, in urban contexts, the fare is the same regardless of the origin-destination pair. Below, we consider two cases: (a) turnstiles at the station entrance that measure only passengers entering, with no indication which direction they will follow; (b) turnstiles upon access to platforms that also give information on trip direction (see Figure 1).

The data collected by turnstiles can be used, with low technological investment, for implementing a monitoring system of the whole metro line, generating information about the passengers on each railway section (between two stations). Such information can be of great use to metro operators for implementing real-time strategies, like a frequency increase or reduction, determination of train composition (number of passenger carriages), the scheduling of additional runs, and so on.

The problem to solve is the estimation of loads on the line starting from turnstile data. For this purpose, we propose feedforward ANNs, which are suitable because (a) the relationships between inputs and outputs do not need to be explicitly known, (b) the results are obtained rapidly, and (c) the boundary conditions do not usually change so much as to invalidate forecasts.

The structure of the ANN provides an input layer with a node for each turnstile, an output layer with a node for each convoy load, and one or more hidden layers. The best structure of the ANN has to be designed for each specific problem. A crucial point concerns the dynamic nature of the problem: the train moves along the line, loading and unloading passengers at stations in different time intervals. Therefore, the number of onboard passengers between two stations at time *t* depends on the passengers loaded and unloaded at previous stations at different times (<*t*). Hence, ANN inputs have to consider turnstile counts referring to several time intervals preceding those being forecast. The number of inputs will depend on the travel time duration between terminals.

The other crucial point is the training phase of ANNs; here we propose to use a supervised learning method, where the example datasets are generated through dynamic simulation models (see Section 4). Note that it is not possible to use real-world data for the training phase. Indeed, only the input data (on passengers entering the stations) are available while the output data (on-board passengers) can be known only if all coaches have sensors that are able to measure them; in this case, however, the proposed approach would not be useful.

## 4. Generation of Training Datasets

To generate the training datasets, we used the simulation model proposed in [65]. This model assumes that:

1. platforms can accommodate all passengers (incoming, waiting and outgoing);
2. at each station and for each direction there is only one platform available;
3. the dwell time is constant and independent of the number of passengers alighting and boarding;
4. there is no interaction on the platform between alighting, boarding and waiting passengers;
5. the capacity of each train is fixed;
6. passengers are distributed uniformly among the train coaches;
7. there is no interaction in the train between alighting, boarding and onboard passengers.

In our test, we assume that the passengers follow a FIFO (First In-First Out) rule for boarding the convoy. The analytical details of the model can be found in [65].

Using this model, we generate the training datasets on the case study as follows: (a) numerous origin-destination (OD) matrices referring to 15′ intervals are randomly generated starting from a base OD matrix; (b) four OD matrices, referring to four consecutive time intervals, are assigned to the metro line, yielding as results the passengers counted at turnstiles, for each interval, and the passengers onboard in each railway section in the next time interval; (c) the output data of the problem are the passengers on railway sections, while the input data are the passengers counted at turnstiles in four time intervals, corresponding to the four previous 15′ periods (e.g., flows on railway sections between 10:15 and 10:30 are estimated according to the turnstile counts in the intervals: 9:15–9:30, 9:30–9:45, 9:45–10:00 and 10:00–10:15). Therefore, the structure of the training datasets is reported in Table 1, where the following notations are used:

- *ds* is the number of datasets;
- *t* is the period under analysis;
- *c^i^_j,t_* is the passenger count at turnstile *j* in period *t* for dataset *i*;
- *f^i^_k,t_* is the load on railway section *k* in period *t* for dataset *i*.

## 5. Case Study and Numerical Results

**Figure 2:**
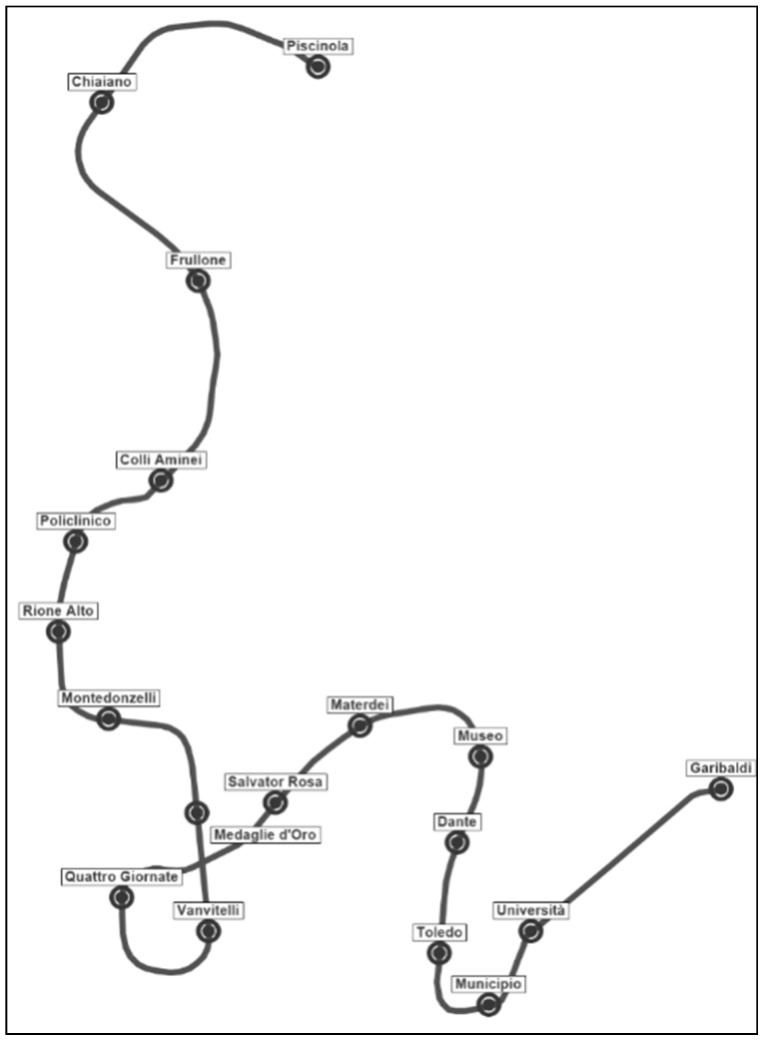
Line 1 route.

The proposed approach was tested on Line 1 of the Naples metro system. This line (see Figure 2) is 18 km long and has 18 stations; it connects high-density districts in Naples and is crucial infrastructure for urban mobility.

Considering these characteristics, we have 34 or 18 turnstiles, if we divide the passengers according to direction or not, and 34 mono-directional railway sections. The main features of the line are summarised in Table 2.

The training datasets were obtained by simulating 2500 OD matrices generated randomly. On eliminating some of them because their results were not feasible (too many passengers compared to the actual capacity), we generated 2279 training datasets. Of the latter datasets, 2229 were used for the whole training process of the ANNs with the software MatLab: 1561 training datasets (70%), 334 validation datasets (15%) and 334 testing datasets (15%). The remaining 50 datasets were used to verify the goodness of the trained ANNs with examples that were not used before in the training process. We tested six ANN structures for both cases: (a) turnstiles at the station entrance (18 turnstiles), and (b) turnstiles at the access points to platforms (34 turnstiles). We thus trained and tested 12 ANNs, as reported in Table 3.

The training phase required computing times from 30 s (case a_1_6) to 8 min (case b_2_20), with a Personal Computer Hewlett Packard i7-7700HQ, 280 GHz, RAM 16 GB. In Table 4 we report the best and worst coefficients of determination (R^2^) for each case, referring to the 50 datasets not used in the training phase, and the corresponding averages and variances. The datasets for which R^2^ is lower than 0.9, 0.8, 0.7 and 0.6 are reported in Table 5. In these tables, the best values for each ANN are underlined.

**Figure 3:**
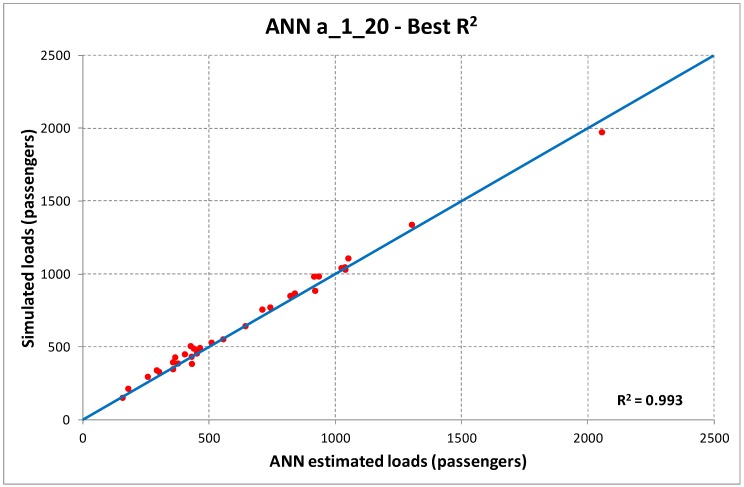

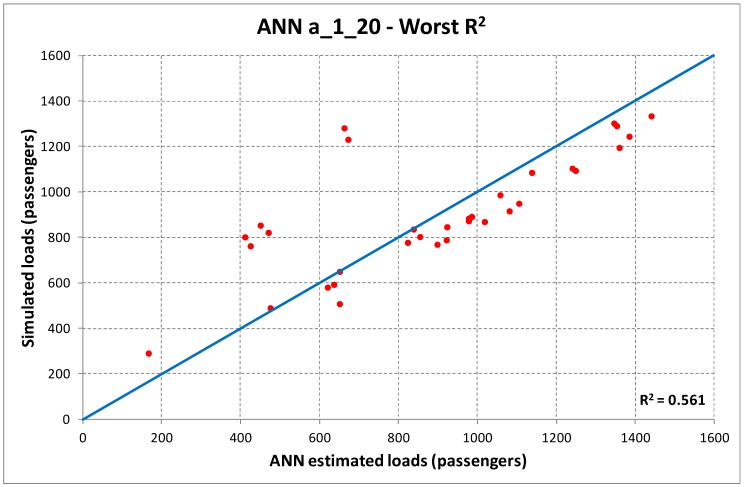
Dispersion diagrams ANN a_1_20 (best, upper diagram; worst, lower diagram).

**Figure 4:**
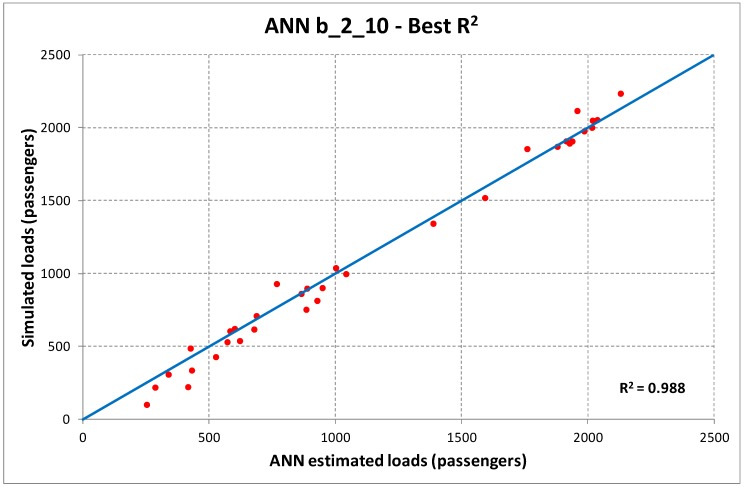

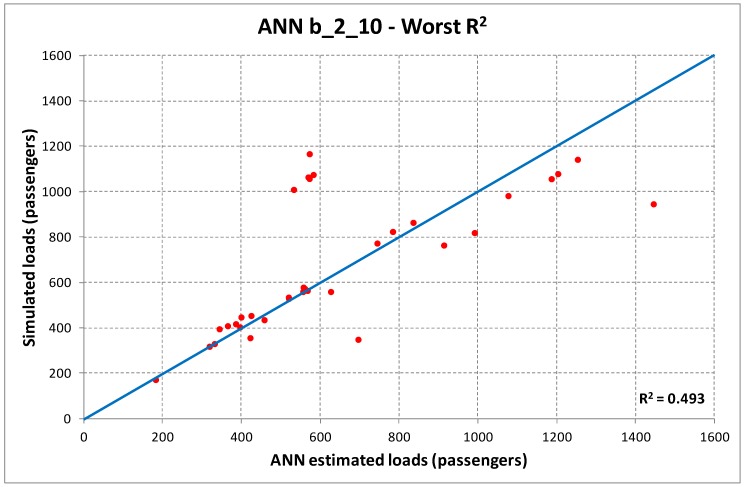
Dispersion diagrams ANN b_2_10 (best, upper diagram; worst, lower diagram).

Examining the results reported in Table 4 and Table 5, we may identify as best ANN structures the one with one hidden layer and 20 neurons for case (a), and two hidden layers and 10 neurons for case (b). The corresponding dispersion diagrams in the cases of best and worst R^2^ are reported in Figure 3 and Figure 4.

## 6. Conclusions and Research Prospects

In this paper, we studied the problem of forecasting passenger flows on railway sections of a metro line starting from counts at turnstiles and proposed to use artificial neural networks (ANNs) for its solution. The training datasets were generated using a simulation model. We considered two cases: turnstiles at station entrances and turnstiles at platform accesses. For both, we designed and trained several ANNs.

The results showed a good capacity of ANNs to forecast the loads on railway sections. Our analysis allowed us to identify the best ANN structure for each case.

Future research could profitably lead in several directions. First of all, other ANN structures could be tested. Then the problem could be extended to more complex metro systems, including systems with more than one line. Finally, other methods could be investigated, as well deep-learning approaches that could be applied to this problem.

## Figures and Tables

**Table 1:** Structure of training datasets for period *t*.

Dataset →	1	2	…	*ds*
*Input data*				
Turnstile 1–Period *t* − 1	*c* ^1^_1,*t−*1_	*c* ^2^_1,*t−*1_	…	*c^ds^*_1,*t−*1_
Turnstile 2–Period *t* − 1	*c* ^1^_2,*t−*1_	*c* ^2^_2,*t−*1_	…	*c^ds^*_2,*t−*1_
…	…	…	…	…
Turnstile *ts*–Period *t* − 1	*c* ^1^ *_ts_*_,*t−*1_	*c* ^2^ *_ts_*_,*t−*1_	…	*c^ds^_ts_*_,*t−*1_
Turnstile 1–Period *t* − 2	*c* ^1^_1,*t*−2_	*c* ^2^_1,*t*−2_	…	*c^ds^*_1,*t*−2_
Turnstile 2–Period *t* − 2	*c* ^1^_2,*t*−2_	*c* ^2^_2,*t*−2_	…	*c^ds^*_2,*t*−2_
…	…	…	…	…
Turnstile *ts*–Period *t* − 2	*c* ^1^ *_ts_*_,*t*−2_	*c* ^2^ *_ts_*_,*t*−2_	…	*c^ds^_ts_*_,*t*−2_
Turnstile 1–Period *t* − 3	*c* ^1^_1,*t*−3_	*c* ^2^_1,*t*−2_	…	*c^ds^*_1,*t*−2_
Turnstile 2–Period *t* − 3	*c* ^1^_2,*t*−2_	*c* ^2^_2,*t*−3_	…	*c^ds^*_2,*t*−3_
…	…	…	…	…
Turnstile *ts*–Period *t* − 3	*c* ^1^ *_ts_*_,*t*−3_	*c* ^2^ *_ts_*_,*t*−3_	…	*c^ds^_ts_*_,*t*−3_
Turnstile 1–Period *t* − 4	*c* ^1^_1,*t*−4_	*c* ^2^_1,*t*−4_	…	*c^ds^*_1,*t*−4_
Turnstile 2–Period *t* − 4	*c* ^1^_2,*t*−4_	*c* ^2^_2,*t*−4_	…	*c^ds^*_2,*t*−4_
…	…	…	…	…
Turnstile *ts*–Period *t* − 4	*c* ^1^ *_ts_*_,*t*−4_	*c* ^2^ *_ts_*_,*t*−4_	…	*c^ds^_ts_*_,*t*−4_
*Output data*				
Railway section 1–Period *t*	*f* ^1^_1,*t*_	*f* ^2^_1,*t*_	…	*f^ds^*_1,*t*_
Railway section 2–Period *t*	*f* ^1^_2,*t*_	*f* ^2^_2,*t*_	…	*f^ds^*_2,*t*_
…	…	…	…	…
Railway section *rs*–Period *t*	*f* ^1^ *_rs_*_,*t*_	*f* ^2^ *_rs_*_,*t*_	…	*f^ds^_rs_*_,*t*_

**Table 2:** Features of Line 1.

Stations	18
Working day runs	241
Convoy capacity (pax/convoy)	864
Line length (km) (outward/return direction)	18.8/18.6
Headway (min)	8–20

**Table 3:** ANN structures.

Case	ANN	Input Nodes	Output Nodes	Hidden Layers	Neurons
a	a_1_6	72	34	1	6
a	a_1_10	72	34	1	10
a	a_1_20	72	34	1	20
a	a_2_6	72	34	2	6/6
a	a_2_10	72	34	2	10/10
a	a_2_20	72	34	2	20/20
b	b_1_6	136	34	1	6
b	b_1_10	136	34	1	10
b	b_1_20	136	34	1	20
b	b_2_6	136	34	2	6/6
b	b_2_10	136	34	2	10/10
b	b_2_20	136	34	2	20/20

**Table 4:** Coefficients of determination (R^2^).

ANN	Best	Worst	Average	Variance
a_1_6	0.9946	0.5487	0.7984	0.0124
a_1_10	0.9941	0.4990	0.8108	0.0160
a_1_20	0.9931	0.5613	0.8332	0.0157
a_2_6	0.9916	0.5353	0.7949	0.0121
a_2_10	0.9930	0.4638	0.8136	0.0175
a_2_20	0.9933	0.5342	0.8244	0.0162
b_1_6	0.9875	0.5460	0.8016	0.0129
b_1_10	0.9905	0.3505	0.8115	0.0202
b_1_20	0.9882	0.4226	0.8291	0.0160
b_2_6	0.9785	0.5119	0.7775	0.0132
b_2_10	0.9889	0.4935	0.8221	0.0132
b_2_20	0.9852	0.4492	0.8075	0.0245

Best values are underlined.

**Table 5:** Analysis of R^2^ values.

ANN	R^2^ < 0.9	R^2^ < 0.8	R^2^ < 0.7	R^2^ < 0.6
Number of datasets				
a_1_6	41	25	9	4
a_1_10	36	22	7	4
a_1_20	33	18	7	3
a_2_6	43	25	10	3
a_2_10	35	21	8	4
a_2_20	34	19	9	5
b_1_6	37	24	10	2
b_1_10	34	23	8	5
b_1_20	32	19	8	3
b_2_6	43	25	10	6
b_2_10	35	20	7	2
b_2_20	30	21	9	7
Percentage of datasets				
a_1_6	82%	50%	18%	8%
a_1_10	72%	44%	14%	8%
a_1_20	66%	36%	14%	6%
a_2_6	86%	50%	20%	6%
a_2_10	70%	42%	16%	8%
a_2_20	68%	38%	18%	10%
b_1_6	74%	48%	20%	4%
b_1_10	68%	46%	16%	10%
b_1_20	64%	38%	16%	6%
b_2_6	86%	50%	20%	12%
b_2_10	70%	40%	14%	4%
b_2_20	60%	42%	18%	14%

Best values are underlined.
